# Accuracy of proton stopping power estimation of silicone breast implants with single and dual‐energy CT calibration techniques

**DOI:** 10.1002/acm2.13358

**Published:** 2021-07-17

**Authors:** Michael S. Chacko, Hardev S. Grewal, Dee Wu, Jagadeesh R. Sonnad

**Affiliations:** ^1^ Oklahoma Proton Center Oklahoma City OK USA; ^2^ Department of Radiological Sciences University of Oklahoma Health Sciences Center Oklahoma City OK USA; ^3^ Department of Radiation Oncology University of Oklahoma Health Sciences Center Oklahoma City OK USA

**Keywords:** breast, dual energy, proton therapy, stopping power

## Abstract

A major contributing factor to proton range uncertainty is the conversion of computed tomography (CT) Hounsfield Units (HU) to proton relative stopping power (RSP). This uncertainty is elevated with implanted devices, such as silicone breast implants when computed with single energy CT (SECT). In recent years, manufacturers have introduced implants with variations in gel cohesivity. Deriving the RSP for these implants from dual‐energy CT (DECT) can result in a marked reduction of the error associated with SECT. In this study, we investigate the validity of DECT calibration of HU to RSP on silicone breast implants of varying cohesivity levels. A DECT capable scanner was calibrated using the stoichiometric method of Bourque et al for SECT and DECT using a tissue substitute phantom. Three silicone breast implants of increasing gel cohesivity were measured in a proton beam of clinical energy to determine ground‐truth RSP and water equivalent thickness (WET). These were compared to SECT‐derived RSP at three CT spectrum energies and DECT with two energy pairs (80/140 kVp and 100/140 kVp) as obtained from scans with and without an anthropomorphic phantom. The RSP derived from parameters estimates from CT vendor‐specific software (*syngo*.via) was compared. The WET estimates from SECT deviated from MLIC ground truth approximately +11%–19%, which would result in overpenetration if used clinically. Both the Bourque calibration and *syngo*.via WET estimates from DECT yielded error ≤0.5% from ground truth; no significant difference was found between models of varying gel cohesivity levels. WET estimates without the anthropomorphic phantom were significantly different than ground truth for the Bourque calibration. From these results, gel cohesivity had no effect on proton RSP. User‐generated DECT calibration can yield comparably accurate RSP estimates for silicone breast implants to vendor software methods. However, care must be taken to account for beam hardening effects.

## 1 INTRODUCTION

A persistent challenge in proton radiotherapy remains the uncertainty of beam range prediction within patient anatomy. A major contributing factor to this proton range uncertainty results from the use of X‐ray computed tomography (CT) images to create proton treatment plans, resulting in total uncertainty of up to 3.5% of the distal range, with the major contributing factor resulting from CT calibration.^1^, ^2^ Proton range is computed from water relative stopping power (RSP) of tissues typically obtained by the user calibration of CT Hounsfield Units (HU’s). However, these calibration methods result in gross errors in RSP computation for prosthetic and implanted devices. Dual‐energy CT (DECT) has shown improvement over single energy CT (SECT)‐derived RSP, especially for such non‐biological materials, especially silicone breast implants.^3^, ^4^

Simple linear characterizations of the relation of HU to RSP have been shown to be inferior to a model‐based approach to characterize HU from elemental compositions to RSP, as proposed by Schneider et al, commonly known as the stoichiometric calibration.^5^ This approach has been adapted for use in DECT, notably by Bourque et al.^6^ The implementation of the stoichiometric calibration with phantoms or materials with properties that deviate significantly from that of biological tissues has been reported to generate uncertainties unacceptable for clinical use.^7^ However, the common practice of using tissue substitutes with elemental compositions similar to biological tissues results in significant errors in derived RSP values for non‐biological materials used in prosthetics or implanted devices; this is especially evident in the case of silicone breast implants where errors in stoichiometric modeled RSP’s have been reported from approximately 13% to 16%.^4^, ^8^

Silicone breast implants have undergone several generations of design philosophy.^9^ Common to all designs, however, is a silicone elastomer shell with a polymer gel of polydimethylsiloxane (PDMS). Innovations in early designs reduced capsular contracture rates and improved shell properties to prevent gel bleed. Later generations further improved shell design and introduced more cohesive polymer gel to prevent gel bleed and migration as well as enabling anatomical shaping of implants. Gel cohesivity, measured by elastic deformation, can be increased by higher cross‐linking of the silicone polymer chains and by hydrogen bonds to “filler” material such as nanoparticles of amorphous fumed silica. The effect of these processes on the properties of finished PDMS gel can only be determined empirically due to differences in gel formation conditions and have an unknown impact on the interactions with protons at energies used for radiotherapy.^10^, ^11^, ^12^, ^13^ To the best of our knowledge, no previous work has examined the potential effect of variable gel cohesivity on proton RSP derived from any CT technique or from direct measurement in a clinical proton beam.

Moyers et al previously reported on directly measured and SECT‐derived RSP’s of silicone breast implants of two prominent manufacturers, Allergan and Sientra.^8^ Empirically measured SECT HU yielded RSP values in their work that deviated approximately +16% from direct RSP measurements, which was shown anecdotally to result in overpenetration in breast treatment plans. This significantly increased heart and ipsilateral lung planned dose. Given the relative uniformity of measured RSP’s across manufacturers, they suggested the use of a manual RSP override to a recommended value of 0.935 for silicone implants contoured in the treatment planning software (TPS). However, since the publication of these results, silicone breast implant manufacturers have introduced new products of varying cohesivity levels. Michalak et al investigated DECT‐based SPR estimation without user CT calibration (e.g., stoichiometric calibration methods) by vendor‐specific software (*syngo*.via, Siemens Healthineers, Erlangen, Germany) that provides direct estimates of the effective atomic number (EAN) *Z_eff_* and relative electron density (RED) *ρ_e_* that can be used to compute the RSP from Bethe's formula.^4^ When applied to a silicone breast implant, they reported a marked improvement in SPR estimation with the DECT method using *syngo*.via over the SECT stoichiometric technique: from 11.45% mean error in RSP to 0.45%. With new products and rapid advancements in breast implant technology, DECT can potentially obviate the need for manual overrides of RSP with values reported in the literature of uncertain relevance across manufacturers and product lines. As noted, however, this method is limited in applicability due to the need for vendor‐specific software. Furthermore, not all TPS allow for direct RSP override or specification but accept the input of specific parameters, such as the mean excitation energy or elemental composition.^14^ Such parameters can be estimated using user‐generated CT calibration. To the best of our knowledge, no previous work has examined user‐generated, vendor agnostic DECT calibration methods on silicone breast implants.

The present work attempts to expand the scope of previously reported recommendations to the treatment of silicone breast implants in proton radiotherapy planning by the inclusion of varied implant types and CT calibration methods. We examine a SECT and DECT stoichiometric approach to HU to RSP calibration on three silicone breast implants of increasing the cohesivity level. These values were compared against computed RSP using the direct extraction of EAN and RED from *syngo*.via software. All computed RSP were finally compared with the direct measurement of RSP with a multi‐layer ionization chamber (MLIC) in a proton beam of clinical energy.

## 2 MATERIALS AND METHODS

Measurements were conducted with an IBA ProteusPLUS proton therapy system (Ion Beam Applications, Louvain‐la‐Neuve, Belgium) and a Siemens SOMATOM CONFIDENCE RT Pro CT scanner (Siemens Medical Solutions USA, Inc.) equipped with the Siemens *syngo*.via software.

The RSP and resulting water equivalent thickness (WET) of the three silicone breast implant samples were directly measured in the clinical proton beam. These values were also derived from SECT and DECT stoichiometric calibrations, as well as *syngo*.via reported values.

The stoichiometric method of Bourque et al parameterizes the scanner HU response of any medium as a function of EAN and RED for SECT calibration, and the EAN as a function of the dual‐energy ratio (Γ) of the low energy HU response (HU_L_) to that of the high energy spectrum (HU_H_). A brief summary of this method follows in *C* and *D*. Two energy pair couples, 80/140 kVp and 100/140 kVp, were calibrated and utilized to assess the RSP estimation robustness across scanner energies in the Bourque calibration method.

### 2.1 Breast implant selection and preparation

Silicone breast implants (Natrelle INSPIRA Round Gel, Allergan, Inc.) were selected with varying gel cohesivity levels and identical volume, as specified by the manufacturer outlined in Table 1.^15^

**TABLE 1:** Breast implant model specifications

Gel	INSPIRA Responsive	INSPIRA Soft Touch	INSPIRA Cohesive
Model	Smooth INSPIRA (SRX)	Smooth Soft Touch (SSX)	Smooth Cohesive (SCX)
Volume (cc)	545	545	545
Cohesivity Level	1	2	3

**FIGURE 1:**
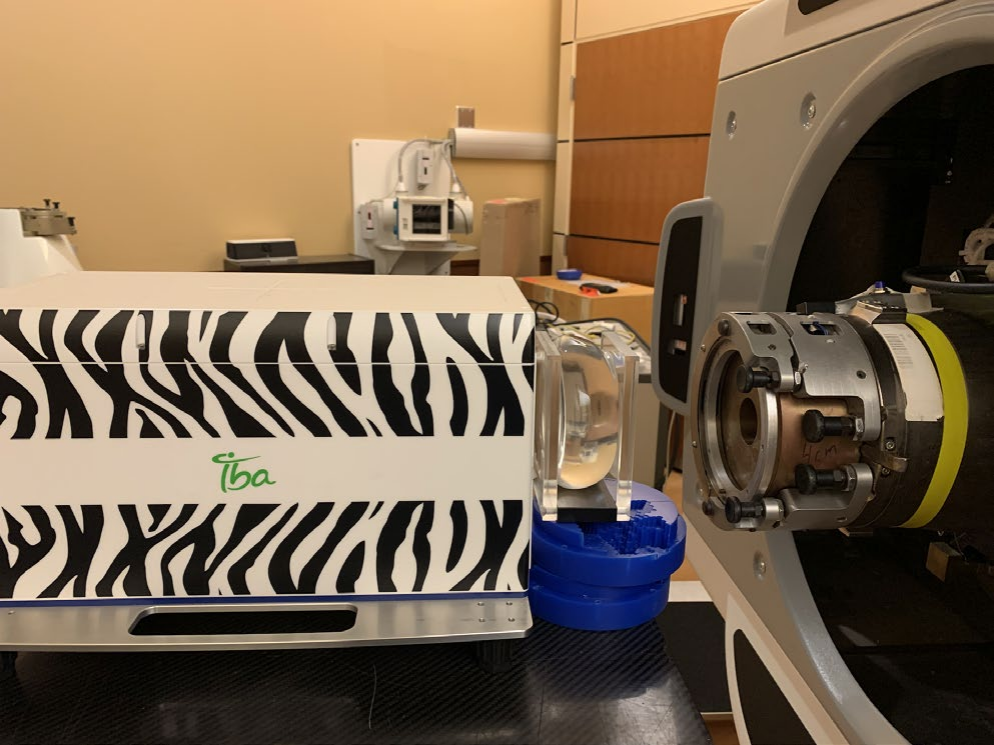
Implant sample affixed between PMMA plates for measurement with the MLIC

For measurements in the proton beam, implants were affixed between two plexiglass plates to maintain a rigid, reproducible shape of uniform thickness (Figure 1).

### 2.2 Proton beam measurement

Direct range measurements of a clinical proton beam that traversed the implant samples were made at the Oklahoma Proton Center. Water equivalent range was evaluated at the distal 90% (R_90_) of the maximum Bragg peak ionization with the use of an IBA Zebra MLIC (IBA Dosimetry), the WET of which was previously calibrated using depth dose measurements made with an ionization chamber in a scanning water phantom.^16^ Breast implant samples were irradiated with a beam of requested R_90_ of 16 cm in water with a field diameter of 6 cm defined at the snout plane by a brass aperture. The range pull‐back through the implants alone was determined by subtracting that of the plexiglass plates with no implant inserted between, resulting in the WET of the implanted sample. The RSP then is the ratio of the WET divided by the physical thickness of the implant on the central axis of the measurement field. Five measurements in the proton beam were obtained for each implant and for the empty plexiglass plates.

### 2.3 SECT calibration

The SECT stoichiometric calibration method of Bourque et al is an adaptation of the method of Schneider, where the model HU response is a product of RED and some function of EAN:

(1) $$u=\rho_{e}f\left(Z_{\mathrm{eff}}\right)$$

where *u* is the scaled HU response

(2) $$u=\;\frac{\left(HU+1000\right)}{1000}\;,$$

for each spectrum used for calibration, and:

(3) $$f\left(Z_{\mathrm{eff}}\right)=\sum_{m=1}^{M}{\hat{b}}_{m}Z_{\mathrm{eff}}^{M‐1}\;.$$

where coefficients ${\hat{b}}_{m}$ is found through empirical fitting during calibration. The degree of polynomial fit $\left(M‐1\right)=5$ was chosen based on the residual analysis of the model HU response.

Tissue substitute plugs in an electron density phantom (Tissue Characterization Phantom Model 467, Sun Nuclear Corporation) summarized in Table 2 were scanned at 80, 100, and 140 kVp with no mAs modulation at 2.0 mm slice thickness in a 20x0.6 mm collimation. The reconstruction kernel Br38 was used for all scans. The HU of each plug was read using a 4.0 cm^2^ region of interest (ROI) along the length of the phantom. The coefficients ${\hat{b}}_{m}$ were found with the least‐squares fitting of the measured HU response using the SciPy Python library.^17^

**TABLE 2:** Tissue substitute plugs used for calibration and testing. Values for RED were obtained from the vendor and are mix specific. Values for EAN and associated uncertainty taken from Bourque et al

Calibration Plug	*ρ_e_*	*Z_eff_*	*ΔZ_eff_*
LN−300 Lung	0.276	7.55	0.03
LN−450 lung	0.432	7.52	0.03
AP6 Adipose	0.924	6.17	0.02
BR−12 Breast	0.96	6.87	0.03
Water Insert	1	7.45	0.02
CT Solid Water	0.99	7.66	0.03
BRN‐SR2 Brain	1.049	6.04	0.03
LV1 Liver	1.062	7.66	0.03
IB Inner Bone	1.082	10.28	0.03
B200 Bone Mineral	1.099	10.29	0.03
CB2‐30% CaCO_3_	1.279	10.76	0.02
CB2‐50% CaCO_3_	1.471	12.4	0.01
SB3 Cortical Bone	1.693	13.51	0.01

Test Plug	*ρ_e_*
Muscle	1.02
CB2‐10% CaCO_3_	1.142

The parameterized HU from the tissue substitute model was plotted against calculated RSP at 200 MeV for 33 biological tissues of known elemental composition from ICRP 23 as utilized in Bourque et al:

(4) $$\mathrm{RSP}=\;\frac{S}{S_{\mathrm{water},\;200\;\mathrm{MeV}}}\;,\;S=\;\rho_{e}\frac{k_{0}z^{2}}{\beta^{2}}\left[\ln\left(\frac{2m_{e}c^{2}\beta^{2}}{I\left(1‐\beta^{2}\right)}\right)‐\beta^{2}\right]\;,$$

where $k_{0}=0.17045\;{\text{MeVcm}}^{‐1}$, z=1, $m_{e}c^{2}=0.511\;\text{MeV}$, $\beta^{2}=0.5662c$ at 200 MeV, and *I* is the mean excitation value.^18^

Interpolation along this curve for measured HU of each breast implant sample yielded the SECT‐derived RSP for each respective CT spectrum calibrated. The resulting WET can be computed by multiplying the RSP by the physical thickness of the separation of the plexiglass plates.

### 2.4 DECT calibration

The tissue substitutes as described in Table 2 were scanned with the Model 467 phantom to acquire HU_L_ = {HU_L,1_…, HU_L,_ *_N_*} and HU_H_ = {HU_H,1_…, HU_H,_ *_N_*}, where *N* = 13 for independent low and high spectrum scans, respectively. Two energy pairs were used: 80/140 kVp and 100/140 kVp with respective dual spiral scans. Both sets were reduced to *u*_L/H_ using Equation 2 and used to compute the dual‐energy ratio, Γ = u_L_ / u_H_ for each of the *N* samples using 4.0 cm^2^ ROI. The EAN can be modeled as a function of Γ and applied to the calibration materials as a linear matrix system:

(5) $$Z_{\mathrm{cal}}=\Gamma_{\mathrm{cal}}c_{k}\;,$$

where *Z_cal_* is an *N* element array of the EAN of the calibration tissue substitutes which were taken from Bourque et al for the Model 467 phantom. The Γ_cal_ is an *N* x *K* array where *K* is determined by the model fit. Fitting coefficients *c_K_* were found using the least squares approach for the tissue substitute materials as in the SECT calibration.

From the model, any material EAN estimation $\left({\hat{Z}}_{\mathrm{eff}}\right)$ using the empirically determined coefficients ${\hat{c}}_{k}$ may be found:

(6) $${\hat{Z}}_{\mathrm{eff}}=\sum_{k=1}^{K}{\hat{c}}_{k}\Gamma^{K‐1}$$

The degree of polynomial fit $\left(K‐1\right)=4$ was chosen based on the residual analysis of model EAN response.

Using the SECT calibration from *C*, the RED of any material can be estimated for individual spectra:

(7) $${\hat{\rho }}_{e,\;L/H}=\frac{u_{L/H\;}}{f(Z_{\mathrm{eff}})}\;,$$

with the function $f(Z_{\mathrm{eff}})$ determined in Equation 3. The final RED estimate (${\hat{\rho }}_{e}$) is computed as the simple average of the RED estimate of each CT spectrum.

The mean excitation energy (*I*) is estimated as a piecewise function of the EAN as originally proposed by Yang et al.^19^ The central region of this function was parameterized based on least squares fit of the human tissue properties in Table 2, resulting in a fifth‐order polynomial function of the EAN as described in Bourque et al where *Z* is equivalent to the EAN:

(8) $$\hat{I}\left(Z\right)=\left\{\begin{array}{cc} e_{1}Z+e_{2} & ifZ<6.26\\ e_{3}Z^{5}+e_{4}Z^{4}\ldots +e_{8} & if6.26<Z<13.52\\ e_{9}Z+e_{10} & ifZ>13.52 \end{array}\right.$$

After parameter estimates ${\hat{Z}}_{\mathrm{eff}}$, ${\hat{\rho }}_{e}$, and $\hat{I}$ were found, the RSP may be computed (Equation 4).

Two tissue substitute plugs not included in the calibration data set, muscle and CB2‐10% CaCO_3_, were scanned in the same phantom with the same technique to verify the correct implementation of the model.

### 2.5 Breast implant CT Scan

Breast implant samples were scanned at one low and high CT spectrum per DECT energy pair. Data were sent to an external workstation with the *syngo*.via software, which provided direct EAN and scaled RED reconstructions for the 80/140 kVp energy pair only. The estimated RED from *syngo*.via can be computed from the scaled RED:

(9) $${\hat{\rho }}_{e,syngo.via}=\frac{{\hat{\rho }}_{e,scaled}}{1000}+1$$

Five scans were obtained of each breast implant sample affixed to the plexiglass plates at 2.0 mm slice thickness, 300 mAs, and reconstructed using the clinically used kernel (Br38). Readings of the resulting HU were obtained using contouring and analysis tools available in RayStation 8B (RaySearch Laboratories). The *I*‐value was computed using Equation 8 as in the DECT stoichiometric calibration and the final RSP from Equation 4.

#### 2.5.1 anthropomorphic phantom measurement

**FIGURE 2:**
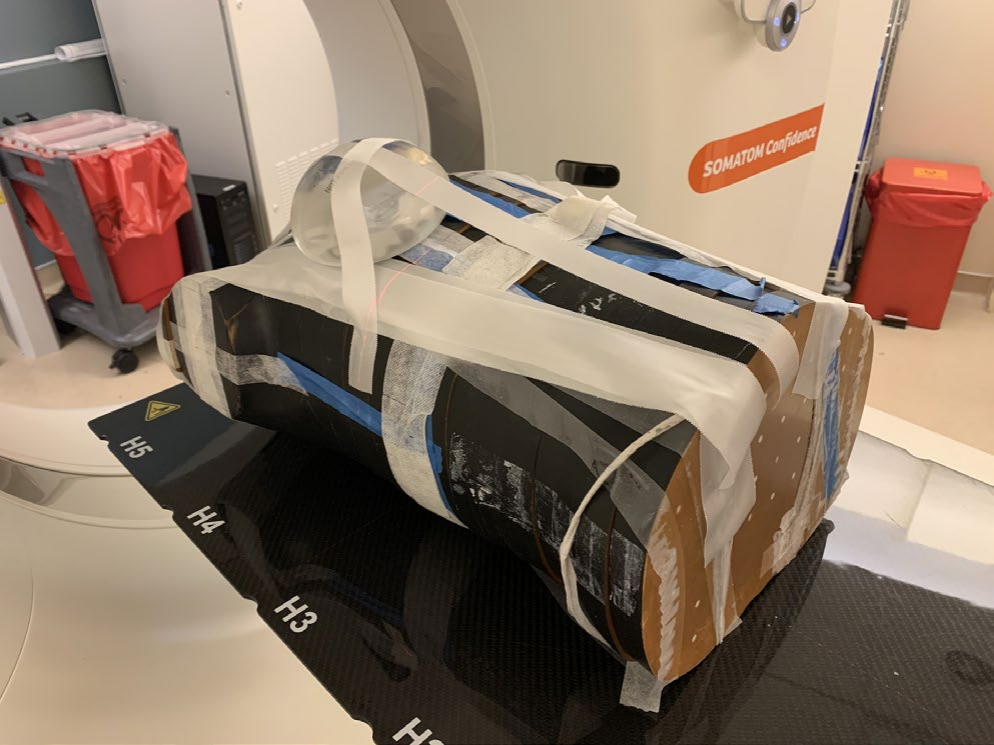
Breast implant sample affixed to Alderson RANDO abdomen phantom

In order to examine the effect of beam hardening on the RSP estimate, the breast implant samples were affixed to an anthropomorphic abdomen phantom (Alderson RANDO, Radiology Support Devices Inc.) (Figure 2) and scanned at 1.5 mm slice thickness, modulated mAs, and reconstructed with the Br38 kernel. The HU were analyzed using RayStation 8B contouring tools. Five scans were obtained for each model implant and processed in *syngo*.via software. The resulting RSP and WET from the stoichiometric calibration was computed in a similar fashion to the implant only setup (non‐RANDO).

## 3 RESULTS

**FIGURE 3:**
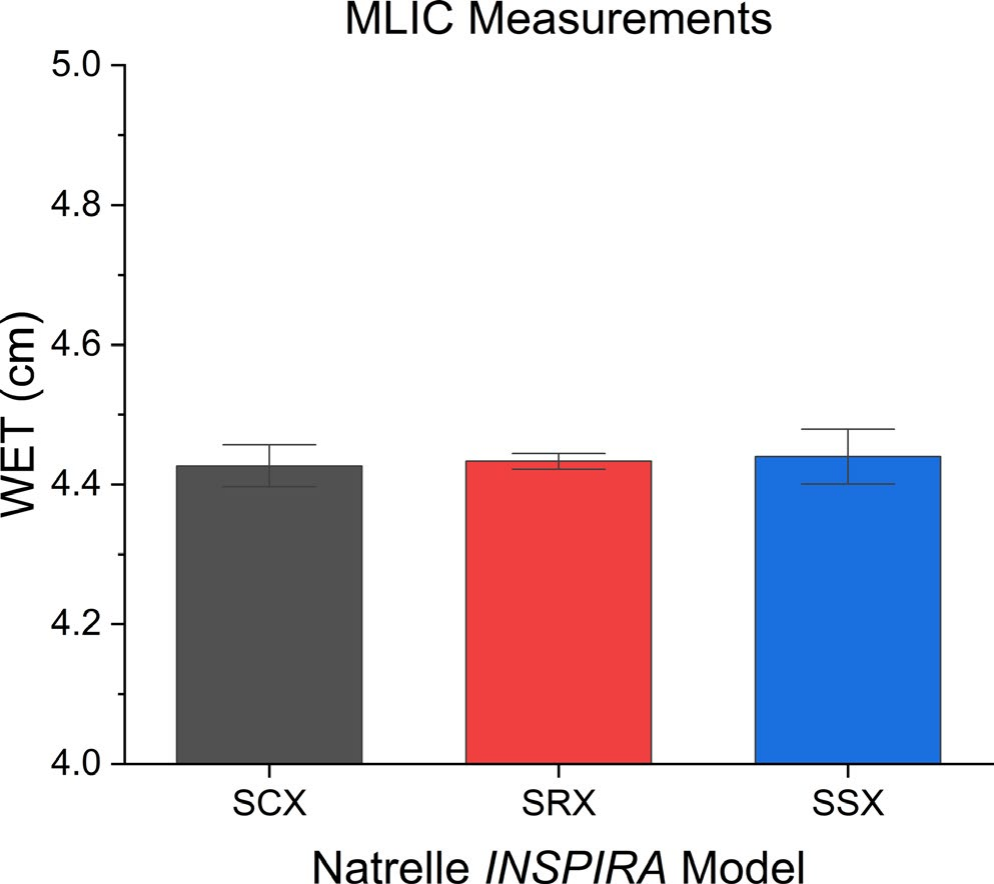
Results of the direct proton beam measurement of WET for model breast implants with the 95% confidence interval

The measured WET from direct proton beam measurement as measured by the MLIC of the three breast implant models is shown in Figure 3 in order of decreasing the cohesivity level (SCX to SSX). No significant difference was found between model cohesivity level and WET (*p* < 0.05).

**FIGURE 4:**
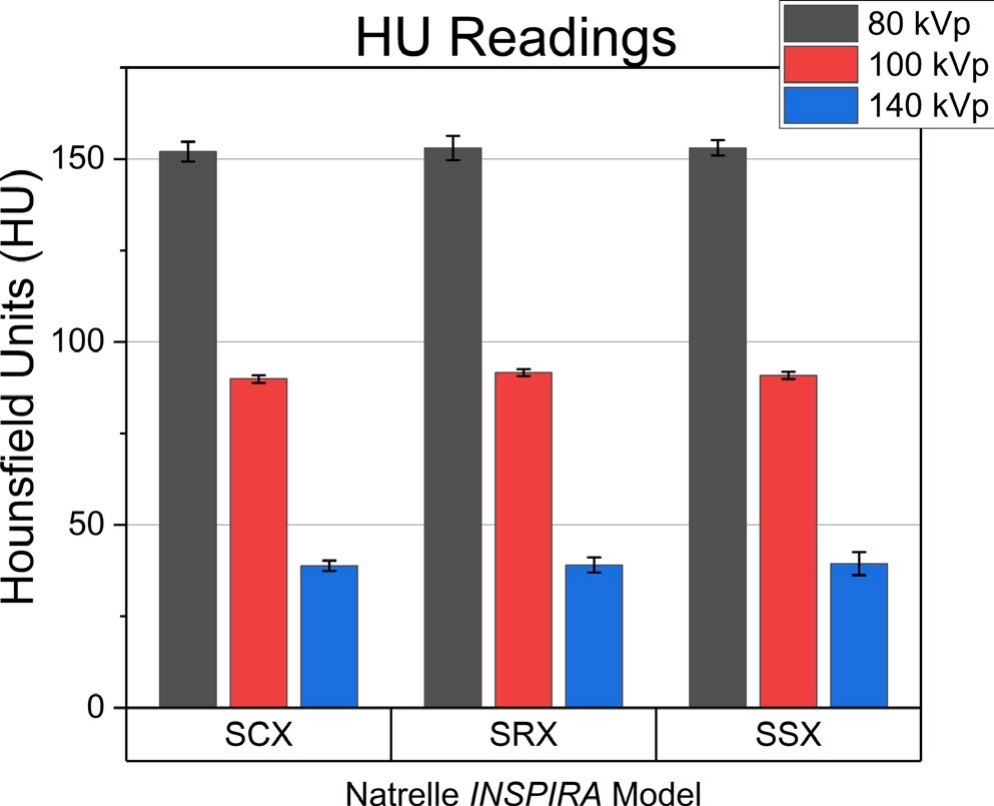
HU of model implants scanned at 80, 100, and 140 kVp SECT on the RANDO phantom

When imaged with SECT, no significant difference (*p* < 0.05) was found between breast implant models measured HU with the inclusion of the anthropomorphic phantom (Figure 4) at any energy.

**FIGURE 5:**
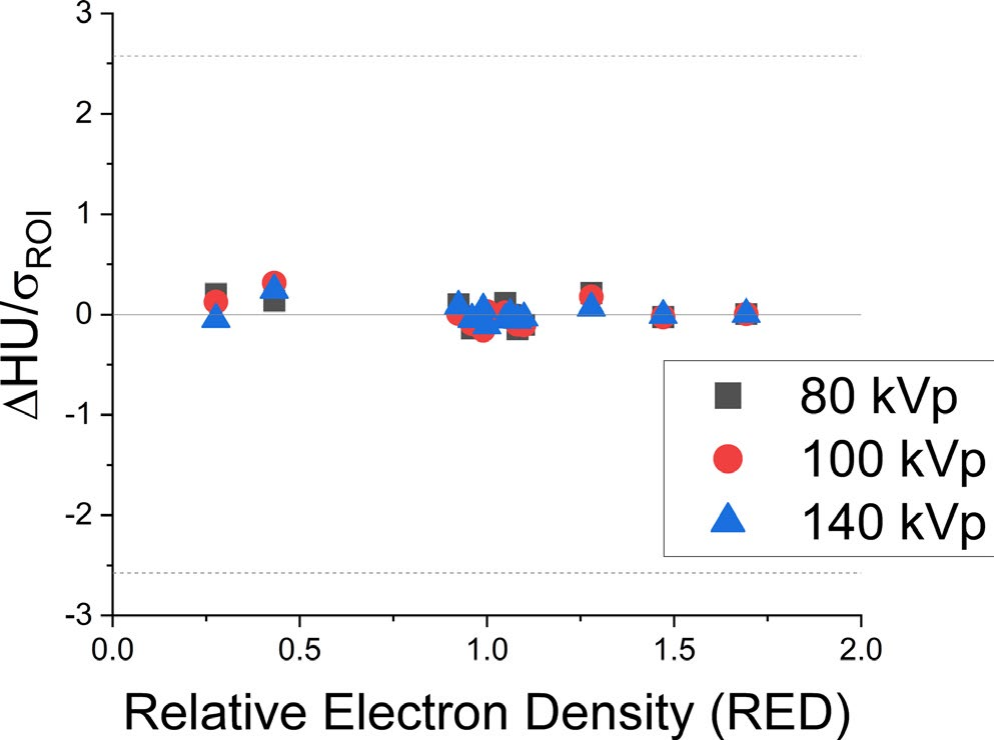
Residual HU (ΔHU) divided by the standard deviation of the ROI for SECT calibration (σ_meas_)

The SECT model fit at *M* = 5 of Bourque et al was assessed by the computed residual of the measured and modeled HU for each tissue substitute plug divided by the standard deviation (σ_ROI_) of HU values of the ROI for each respective plug (Figure 5). All residuals were within one standard deviation for each energy spectrum calibrated.

**FIGURE 6:**
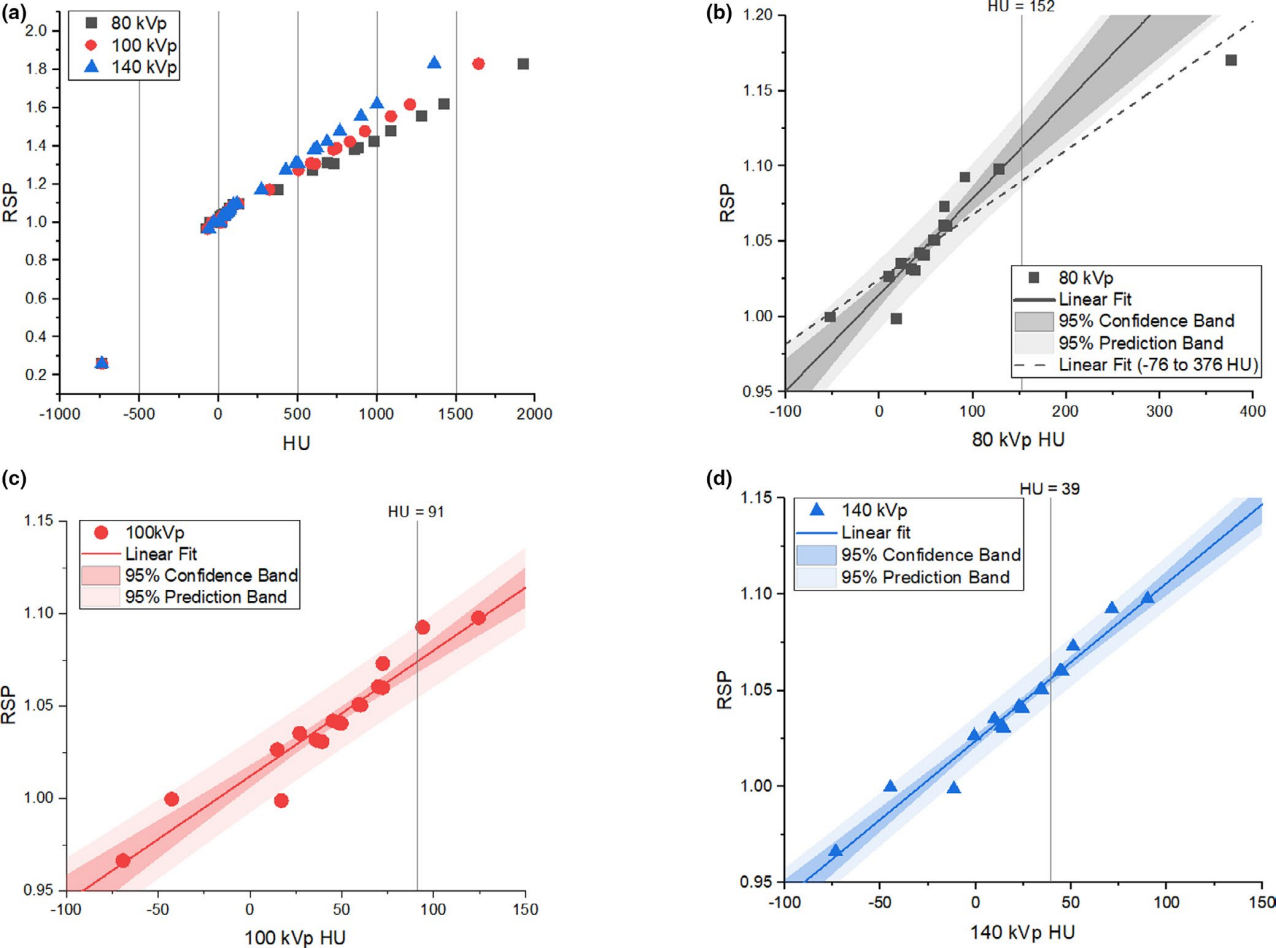
SECT calibration of HU to RSP for 80, 100, and 140 kVp over entire HU range (a), in the central region with linear fit (b‐d). Light and dark bands indicate the 95% prediction and confidence bands, respectively. The 80 kVp linear fit with the inclusion of sacrum bone (HU = 324) is shown in (b) in the dotted line

Figure 6a shows the fit of the model HU response to the computed RSP for 33 human tissues as specified in ICRP 23 for 80, 100, and 140 kVp SECT calibrations. Figure 6b–d show linear fits for the central HU region corresponding to 21 tissue types. The corresponding coefficients of determination increased and the 95% confidence and prediction bands narrowed as spectrum energy increased, indicating higher degrees of linear correlation between HU and RSP. Vertical lines indicate the average measured HU across all model breast implants on the RANDO phantom for each respective energy. At 80 kVp, the measured breast implant HU extended beyond the linear fit region to in between the model HU response of pancreatic tissue (103 HU) and sacrum bone (324 HU). The dotted line (Figure 6b) indicates the fit including sacrum bone, representing an approximate −2% change in RSP at 152 HU.

**FIGURE 7:**
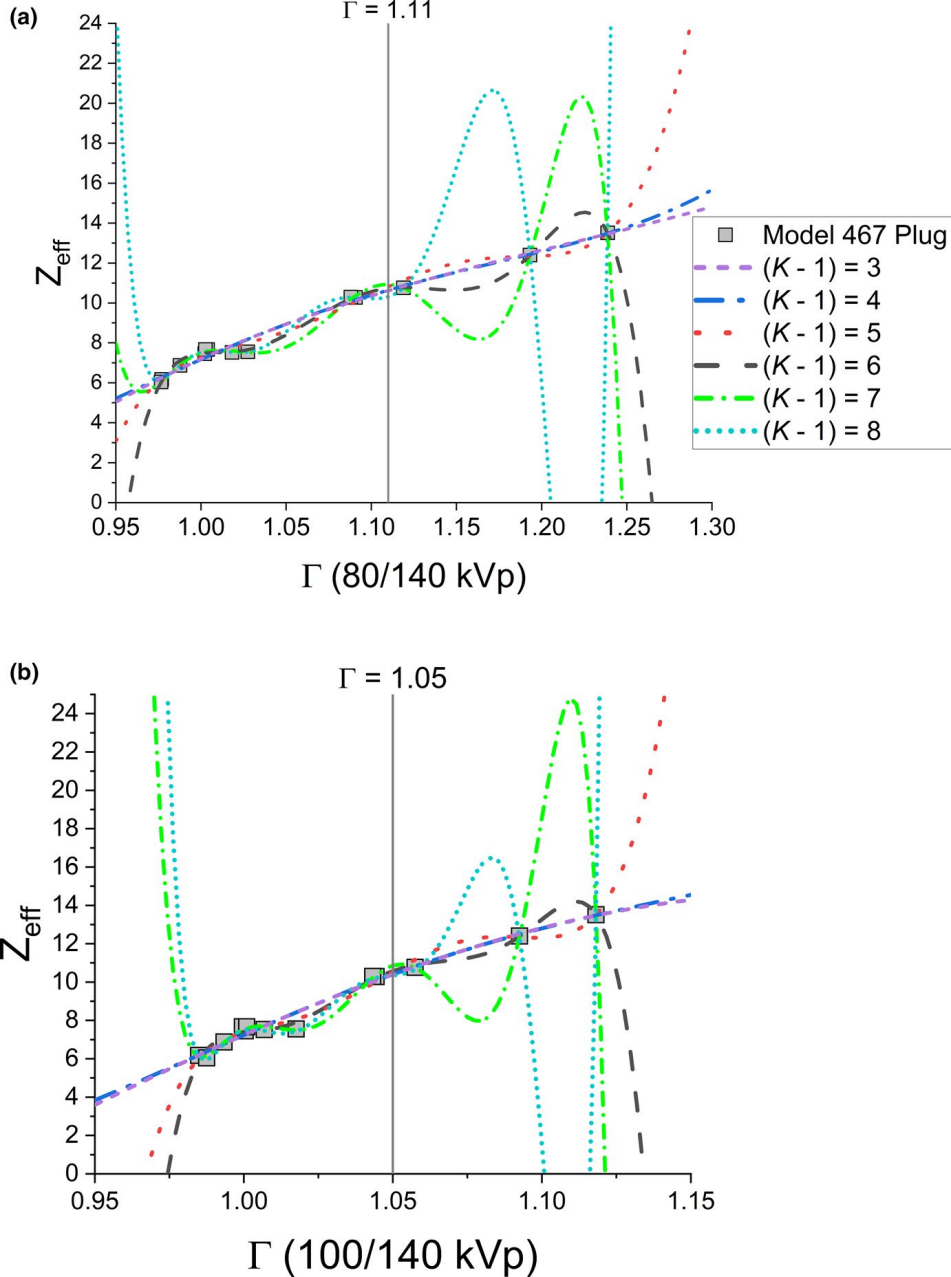
Polynomial fitting of dual‐energy ratio (Γ) to the EAN (*Z_eff_*) for the 80/140 kVp energy pair (a) and 100/140 kVp pair (b). Vertical lines indicate measured Γ averaged over all model breast implants

**FIGURE 8:**
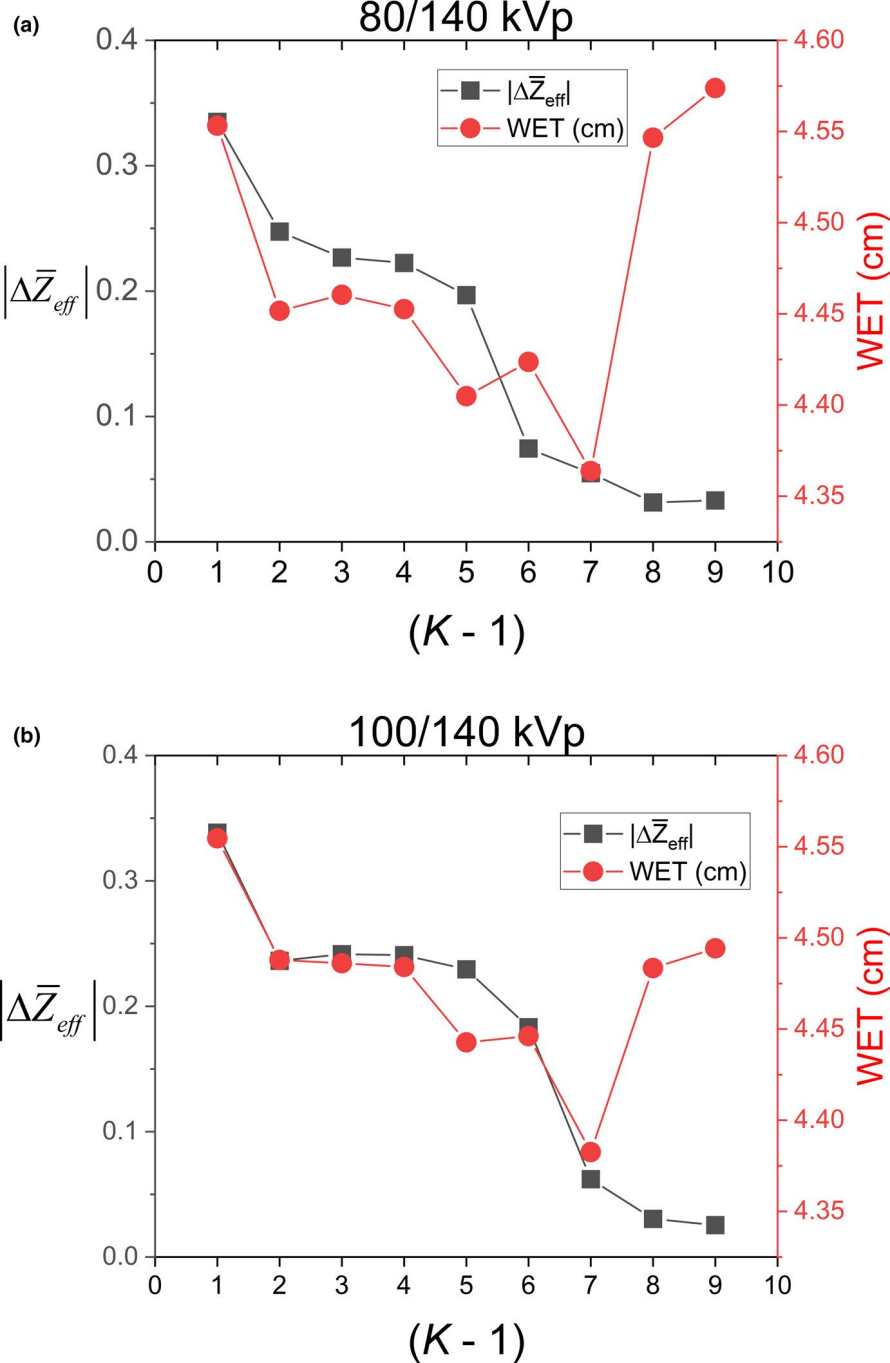
Degree of polynomial fit (K – 1) versus average absolute residual of EAN $\left(\left|\Delta {\bar{Z}}_{\mathrm{eff}}\right|\right)$ overlaid on average WET of model breast implants for DECT energy pairs 80/140 kVp (a) and 100/140 kVp (b)

**FIGURE 9:**
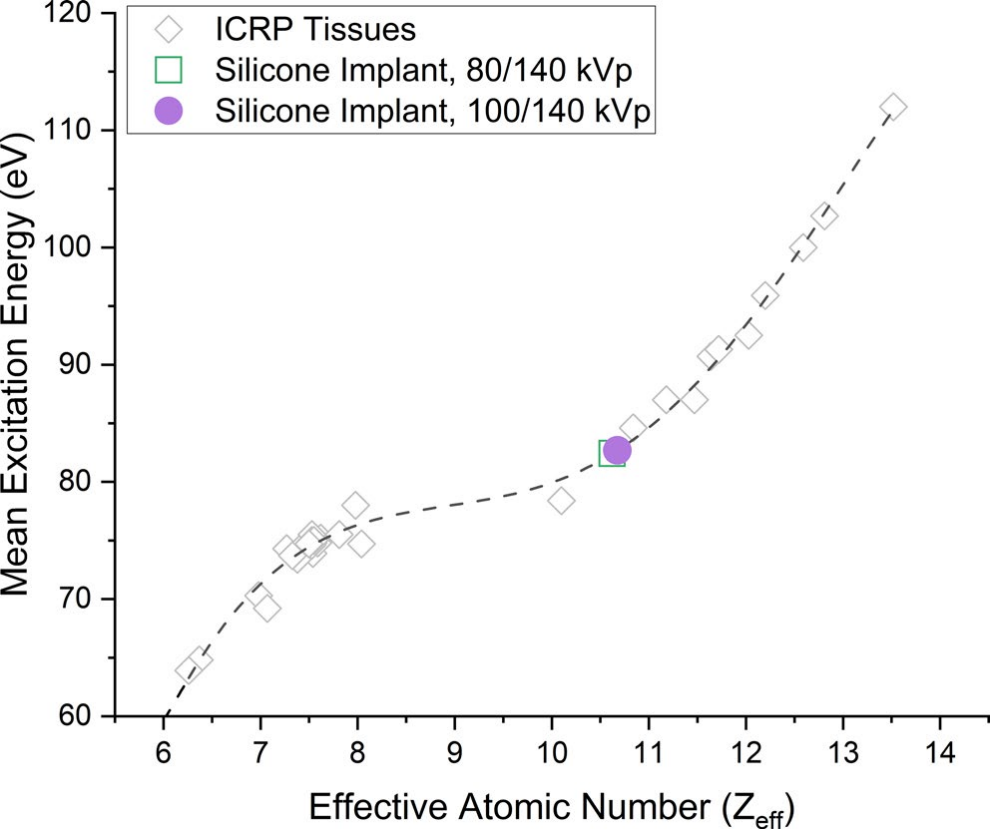
Relationship of EAN to the estimated mean excitation energy $\hat{I}$. Shown are the human tissue data and overlaid are the estimated parameter values for all model silicone implants at both DECT energy pairs

The Bourque calibration for DECT was fit by analyzing the residual absolute difference between modeled and expected EAN of the tissue substitute plugs. A polynomial degree (Equation 6) of *K* – 1 = 4 minimized the residual difference in EAN without overfitting the model (Figure 7 and Figure 8). The parameter $\hat{I}$ from the EAN in Equation 8 averaged over all model silicone breast implants for both DECT energy pairs is shown in Figure 9.

**FIGURE 10:**
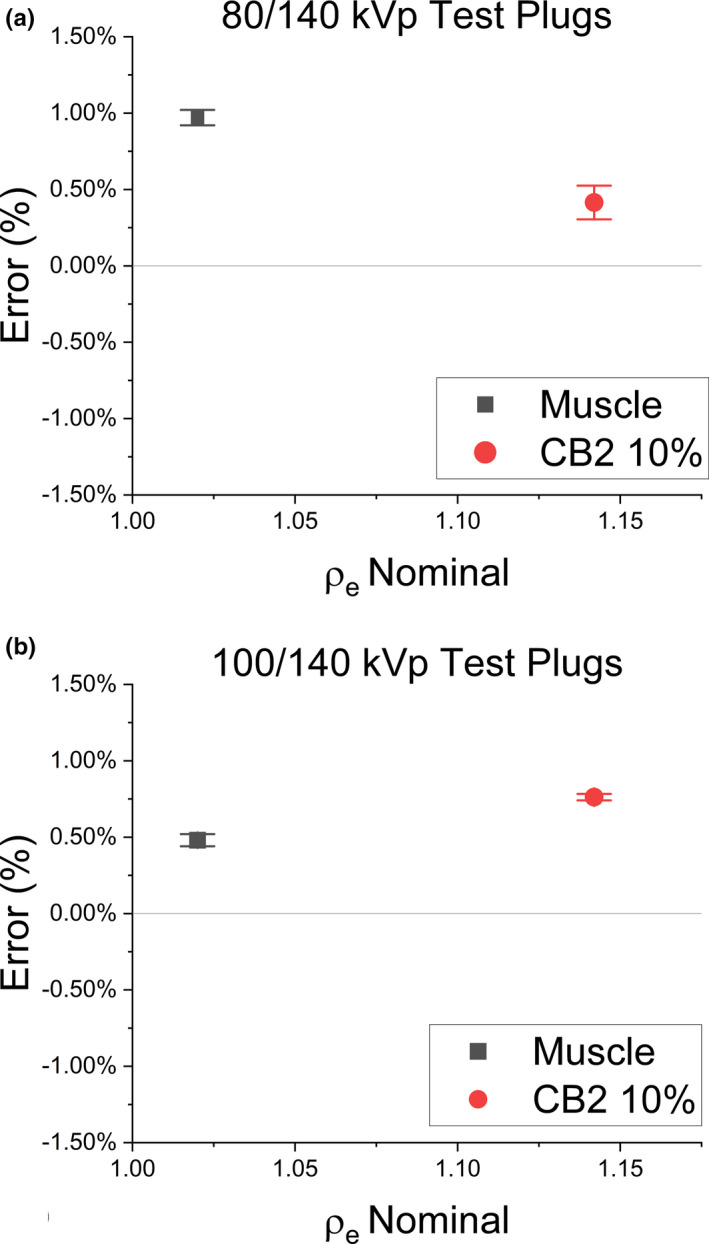
Validation of DECT calibration model implementation using supplied muscle and CB2‐10% plugs for 80/140 kVp (a) and 100/140 kVp (b) in the Model 467 phantom. Error bars indicate the 95% confidence interval of the non‐statistical component

The error in the derived RED for the two test tissue substitute plugs was assessed for each DECT energy pair. For both the muscle and CB2‐10% plugs, the error was ≤1% from vendor‐supplied specifications of RED (Figure 10). The uncertainty of the RED estimate followed the derivation of Bourque et al, using specified Δ*Z_eff_* = 0.02 for muscle tissue and a conservative estimate of Δ*Z_eff_* = 0.03 for CB2‐10%.

**FIGURE 11:**
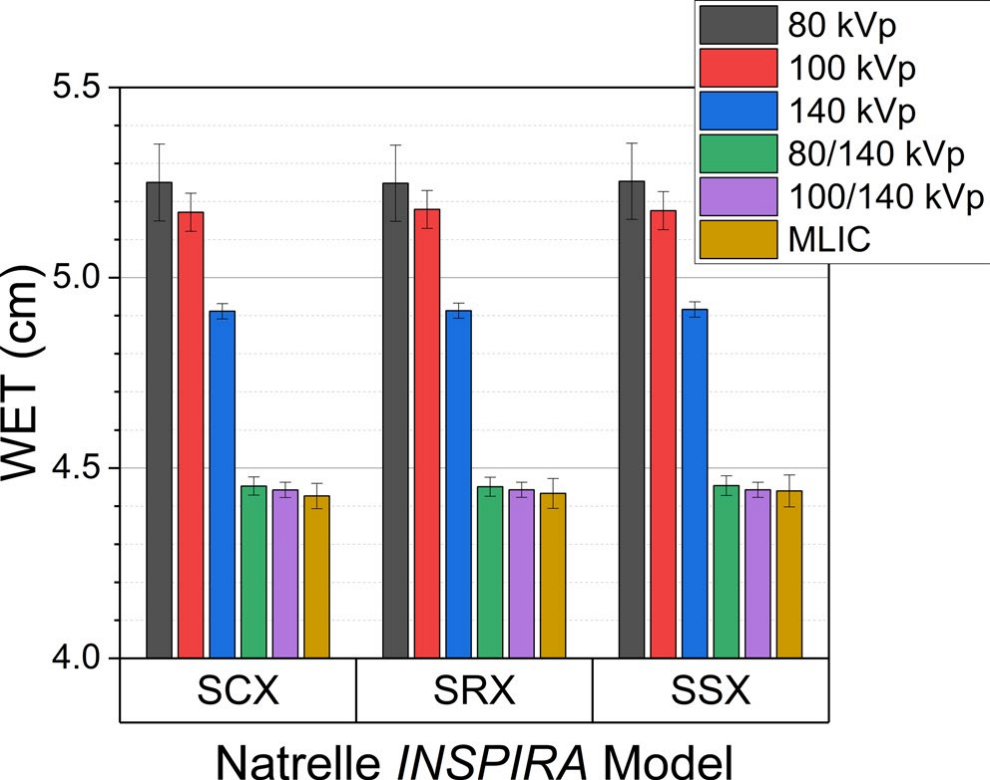
Measured and SECT/DECT‐derived WET for all model breast implants. Error bars are the 95% confidence interval from statistical variation only

**FIGURE 12:**
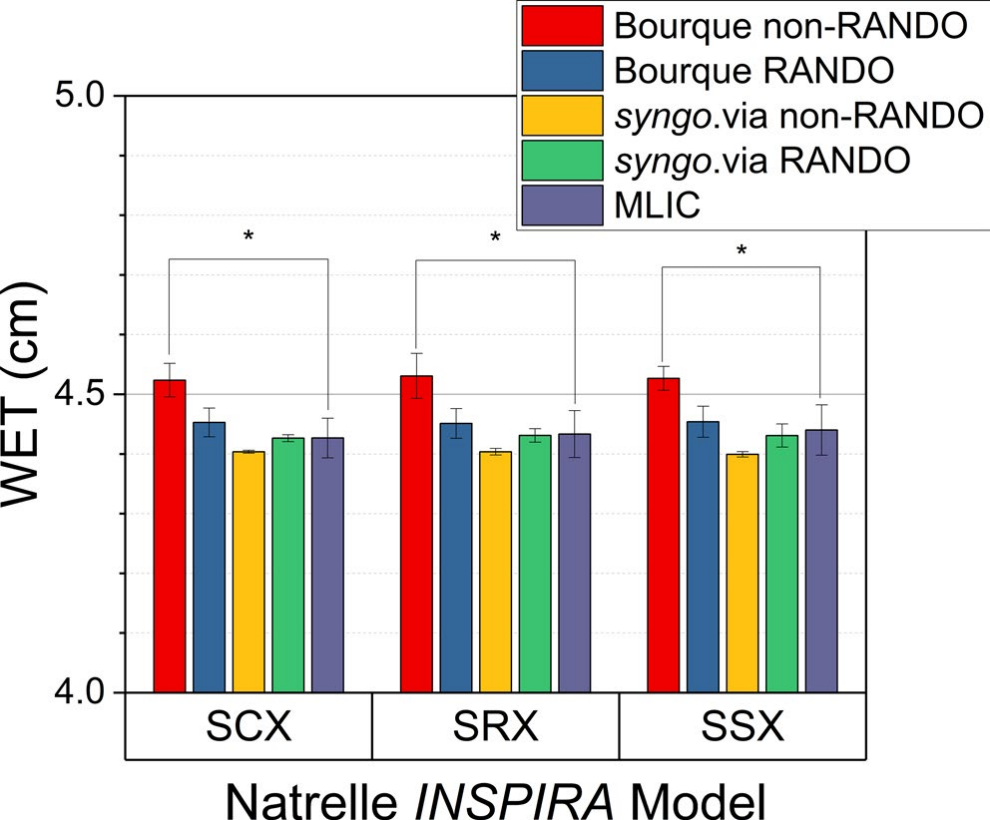
Measured and CT‐derived WET estimates (cm) at 80/140 kVp with (RANDO) and without (non‐RANDO) the anthropomorphic phantom, * indicates a significant difference

When applied to the breast implant samples as measured on the RANDO phantom, the SECT calibrations decreased in error with reference to the ground‐truth MLIC measurements as CT spectrum energy increased as seen in Figure 11. The error of the 80 kVp derived WET estimation of approximately +19% decreased to approximately +17% at 100 kVp, and +11% at 140 kVp. With DECT using the Bourque calibration, the WET estimation error decreased to <0.5% for both energy pairs and was not significantly different from ground‐truth. Similarly, the *syngo*.via WET estimate was within ≤0.1% and not significantly different from the ground‐truth measurements (Figure 12). No significant difference was found between the predicted WET from the Bourque calibration or *syngo*.via (*p* < 0.05) between cohesivity levels for either DECT energy pair.

For the five sample scans taken, the average EAN and RED obtained from the Bourque calibration was 4.8% and 0.8% higher than the *syngo*.via estimate, respectively, averaged over all model breast implants as seen in Table 4 for the 80/140 kVp DECT energy pair. However, the RED has a linear relationship with RSP and is the dominant term, whereas the EAN’s impact is mediated through the ln(*I_med_*) term in Equation 4.

Comparing the DECT‐derived WET from the RANDO phantom scan to the implant and holder alone (non‐RANDO), the Bourque calibration WET estimate decreased by approximately 0.74 ± 0.04 mm averaged over all models, while the *syngo*.via WET estimate increased by approximately 0.03 ± 0.01 mm as seen in Figure 12. Without the RANDO phantom, the Bourque calibration WET estimate was significantly different from the ground‐truth MLIC measurements (*p* < 0.05). The inclusion of the RANDO phantom to the scan in both cases improved the DECT‐derived WET estimate closer to the MLIC ground‐truth estimate.

## 4 DISCUSSION

In proton radiotherapy, accurate computation of proton range with minimal uncertainty has important dosimetric implications for patient treatment. A major source of this uncertainty comes from the calibration of CT HU to proton RSP: the photon‐specific interactions of the imaging system must be translated to charged particle interactions that comprise stopping power. This calibration is generated through the use of tissue substitute materials. As a result, non‐biological material components of prosthetic or implanted devices may not be well characterized using conventional calibration techniques using SECT. Breast implant use in breast reconstruction surgery has historically increased following a mastectomy or lumpectomy in the United States.^20^ In addition, recent generations of breast implant design have enabled selection based on the desired aesthetic outcome by the introduction of variable cohesivity in product portfolios, such as the Natrelle *INSPIRA* line by Allergan Medical.^15^ Our clinical practice has been to manually assign the RSP of silicone breast implants in the TPS based on values reported in the literature; however, these values of RSP were measured using breast implant models that were uncharacterized with regards to gel cohesivity.

As evidenced from Figure 3 and Table 3, increasing gel cohesivity did not correlate with change in proton RSP as measured with a clinical proton beam with a water‐equivalent range of 16 cm. Any variations in the amount or composition of filler materials introduced to increase hydrogen bonds did not significantly alter proton RSP for the breast implants. Moreover, increased polymer cross‐linking does not introduce new chemical species, which would influence the EAN or RED.^13^ The RSP of direct measurements is consistent with the value recommended by Moyers et al of RSP =0.935 for all models tested.^8^

**TABLE 3:** MLIC measured RSP of model breast implants

MLIC Measured RSP		
SCX	SRX	SSX
0.933 ± 0.006	0.933 ± 0.002	0.935 ± 0.008

Proton treatment plans for breast radiotherapy routinely employ *en face* beam arrangements. The clinical implementation of SECT‐derived RSP at any kVp would result in the overpenetration of the breast by the treatment beam as a result of the overestimation of the RSP and WET. As the energy of the SECT spectrum increased, the estimated error in RSP decreased. With an estimated EAN of *Z_eff_* > 10 as determined by DECT analysis (Table 4), the influence of a higher photoelectric component of linear attenuation, with its Z^3^ dependence, at lower CT spectrum energies increases the HU and can potentially increase it outside the range of soft tissue (Figure 6b). In the skeletal bone region, the relation between HU and RSP characterized by the slope decreases rapidly from that of soft tissue.

**TABLE 4:** Parameter estimates, EAD (A) and RED (B) from the Bourque calibration and *syngo*.via software from the 80/140 kVp DECT. Errors represent the 95% confidence interval from the statistical and non‐statistical components

Model	Bourque		*syngo*.via	
	${\hat{Z}}_{\mathrm{eff}}$	ρ_e_	${\hat{Z}}_{\mathrm{eff}}$	ρ_e_
SCX	10.62 ± 0.12	0.948 ± 0.006	10.14 ± 0.06	0.940 ± 0.005
SRX	10.63 ± 0.09	0.948 ± 0.006	10.13 ± 0.06	0.941 ± 0.005
SSX	10.62 ± 0.09	0.948 ± 0.006	10.14 ± 0.08	0.941 ± 0.005

Dual‐energy computed tomography (DECT) has the potential to improve proton range estimation from CT data.^21^, ^22^, ^23^ The interactions of the photon CT imaging beam and the proton treatment beam with patient anatomy are both inherently reliant upon the RED, but proton stopping power is also dependent on EAN and the mean excitation value of the medium (*I_med_*) as described in the Bethe formula. The EAN and *I_med_* are defined by the elemental compositions of the medium, but this relation is not equivalent to that of CT HU values, as modeled in the stoichiometric calibration. As evidenced by HU degeneracy with respect to RSP, these fundamental differences in photon and proton interactions can lead to increased uncertainties in the SECT stoichiometric calibration.^24^ Proposed DECT‐based approaches for HU to RSP conversion include the estimation of EAN, *I_med_*, and RED, which then can be used directly in the Bethe formula though the methods of extraction of these parameters differ.^22^ Most require user calibration of the CT scanner by the use of tissue substitutes as in the case of SECT stoichiometric calibration. In particular, the method of Bourque et al models the HU of any medium as a function of EAN and RED, while obtaining *I_med_* from a fifth‐order polynomial fit from the EAN.^6^ This method introduces a SECT stoichiometric calibration that simplifies the two‐parameter empirical fit of *Z* described in Schneider et al with respect to the photoelectric and Compton components of the linear attenuation coefficient to a single polynomial function of *Z_eff_* of variable order *M* ‐ 1 and coefficients *b_m_* obtained by least‐squares fitting of tissue substitute materials. Coefficients are fit for the low energy and high energy of the dual‐energy pair separately and used to estimate the RED by simple averaging. The EAN is modeled as a *K*‐*1*‐order polynomial function of either a dual‐energy ratio or index Γ. Ex vivo validation of the method by Xie et al demonstrated marked improvement over SECT stoichiometric SPR estimation.^25^

From our results, both the user‐generated and vendor extracted parameters yielded WET estimates well within distal range uncertainty margins proposed in the literature, typically on the order of 3.5%.^24^ The accuracy of the Siemens *syngo*.via parameter estimates also is in good agreement with previously reported values by Michalak et al of approximately ≤1.1% in a non‐anthropomorphic experimental setup.^4^ This suggests that the use of DECT calibration can obviate manual overrides for silicone breast implants for treatment planning, even when implemented with a user‐generated parametrized EAN/RED model. Although such models have demonstrated difficulty in modeling the photoelectric effect in human tissues, such as the thyroid, the similar results at both DECT energy pairs indicate that PDMS can be well characterized. The Bourque calibration EAN estimate agrees better than *syngo*.via with the referenced value for silicone of Z_eff_ ≈ 10.7; however, the specific composition may vary with the manufacturing process.^11^, ^26^ Both models agreed better with respect to RED, which is directly linear with RSP as specified in the Bethe–Bloch equation.

The use of polynomial fitting for DECT calibration, while allowing continuous relationships with EAN, poses unique challenges in regions of sparse data. Higher‐order polynomial fits can lead to overfitting, where the residual EAN values of calibration materials are minimal, but gross errors may result for materials at extremes or in the bone region.^25^ The dual‐energy ratio for silicone breast implants was relatively robust with respect to polynomial fit; however, we utilized the lowest degree of the polynomial fit to simulate a clinically implemented model. The use of monotonically increasing fitting coefficients as demonstrated by Xie et al is recommended for clinical utilization of the Bourque calibration model.^25^

From Figure 12, the inclusion of the RANDO phantom improved DECT estimates of WET for both techniques used. In both SECT and DECT, user‐generated calibrations, a significant source of variability in RSP estimates comes from the difference in the size of the calibration medium and the subject of RSP estimation arising from varying amounts of beam hardening.^25^, ^27^ As subject size becomes larger, increased beam hardening of the CT spectrum should result in relatively lower attenuation and consequently lower RSP as seen in the Bourque calibration RSP estimate, but the *syngo*.via RSP estimate marginally increased. This indicates that the *syngo*.via model is more robust for variations in beam hardening with respect to subject size than user calibration using phantoms. The DECT stoichiometric approach has been reported to have equal or higher variation in derived RSP with variable calibration phantom diameter than SECT calibrations.^25^ It is important to note that this effect likely extends to all materials, beyond that of the silicone breast implants examined in this work. When evaluating the CT calibration in the clinic for uncharacterized or implanted materials, it is therefore important to account for beam hardening effects in the experimental setup.

Limitations of this work included limited energy selection associated with *syngo*.via in DECT on the scanner used in this study; higher energy pairs could not be evaluated for comparison. Alternate DECT calibration methods or tissue substitute materials could also be explored for robustness when applied to non‐biological materials.

## 5 CONCLUSIONS

Accurate computation of proton RSP is an essential component of minimizing treatment plan uncertainty. With respect to silicone breast implants, variations in gel cohesivity within a product line do not have a significant impact on proton RSP. As breast implant technology advances, direct estimation of RSP can be improved through the use of DECT. A user‐generated stoichiometric DECT calibration can potentially yield RSP estimates well within institutional range uncertainty margins. Vendor‐specific parameter extraction methods, however, can be more robust with regards to beam hardening‐related variations in derived RSP.

## CONFLICT OF INTEREST

None to report.

## AUTHOR CONTRIBUTIONS

MC designed and carried out the experiment. HG helped carry out the experiment and reviewed the results. DW and JS reviewed all results and manuscripts. MC wrote the manuscript with input from all authors.

## Data Availability

The data that support the findings of this study are available from the corresponding author upon reasonable request.
